# ﻿*Violashiweii*, a new species of *Viola* (Violaceae) from karst forest in Guizhou, China

**DOI:** 10.3897/phytokeys.196.83176

**Published:** 2022-05-16

**Authors:** Xiao-Chen Li, Zheng-Wei Wang, Qi Wang, Bin-Jie Ge, Bin Chen, Ping Yu, Xin Zhong

**Affiliations:** 1 Eastern China Conservation Center for Wild Endangered Plant Resources, Shanghai 201602, China; 2 Shanghai Chenshan Botanical Garden, Shanghai 201602, China; 3 Guizhou Maolan Karst Forest, Libo 558400, China

**Keywords:** Morphology, phylogeny, sect. *Plagiostigma*, subg. *Viola*

## Abstract

*Violashiweii* Xiao C. Li & Z. W. Wang (Violaceae), a new species from Guizhou, China, is described, based on morphological and molecular evidence. The new species is morphologically most similar to *V.kwangtungensis* Melchior in its glabrous lateral petals and stoloniferous habit, but can be distinguished by its obtuse teeth along the leaf margin, its abaxially greyish-green leaf blade and its broader and entire sepals with a distinct basal appendage.

## ﻿Introduction

*Viola* L. is the largest genus amongst the Violaceae, comprising approximately 580–620 species (Wahlert et al. 2014; Marcussen et al. 2015), which are widely distributed in temperate regions and tropical high mountain regions worldwide, with south-western China as one of its current centres of diversity (Wahlert et al. 2014). The diversity and high number of species has resulted in an extremely complex interspecific relationship in this genus due to hybridisation and horizontal evolution amongst sections and species (Marcussen et al. 2015). Since Becker (1925) provided the first infrageneric classification for *Viola*, several infrageneric classifications of the genus have been proposed (Clausen 1927, 1929, 1931, 1964; Gershoy 1934). In the latest taxonomical revision of *Viola* of China, 96 species were recognised as native (Chen et al. 2007). However, delimitation of the species with stolons distributed in southern and south-western China remains highly problematic and new species are still being discovered (Zhou and Xing 2007; Chen and Yang 2008, 2009; Zhou et al. 2008a; Dong et al. 2009; Ning et al. 2012; Huang et al. 2021).

During an expedition to Guizhou Province in November 2019, an unfamiliar violet whose habit was somewhat similar to that of *Violakwangtungensis* Melchior caught the authors’ attention on the karst rock outcrops. Several specimens with cleistogamous flowers were collected from the field and living material was transplanted and cultivated in Chenshan Botanical Garden for further observation.

## ﻿Materials and methods

In this study, molecular phylogenetic analysis, based on the ITS dataset, was firstly conducted to resolve the phylogenetic position of the unfamiliar violet and its relationship with *V.kwangtungensis* Melchior. Subsequently, morphological characters of this unfamiliar violet and its related species were compared, based on living plants and herbarium specimens, including the digital resource of the Chinese Virtual Herbarium (https://www.cvh.ac.cn/) and the China Field Herbarium (https://www.cfh.ac.cn/). Herbarium specimens were examined in IBK and CSH. Original protologues and relevant literature were also investigated. Leaf material of the putative new species and its related species was collected and stored with silica. Six species, represented by eight individuals, were newly sampled. Voucher specimens were deposited in Chenshan Herbarium (CSH). Total genomic DNA was extracted with the modified CTAB method (Doyle and Doyle 1987) for library construction at Benagen (https://www.benagen.com). Paired-end sequencing of the whole sequences from both ends of 150 bp fragments was performed on the DNBSEQ T7, about 2 Gb clean data for every sample were produced. The nrDNA were de novo assembled using the GetOrganelle pipeline (Jin et al. 2020) and sequences of ITS1-5.8s-ITS2 were extracted with ITSx 1.1.3 (Bengtsson-Palme et al. 2013). Another 31 sample sequences were obtained from NCBI (Gong et al. 2010; Liang and Xing 2010). The sequences of the species and related ones were aligned in Geneious Prime 2021.2.2 (https://www.geneious.com) using MAFFT (Katoh and Standley 2013) by default setting. Phylogenetic construction was conducted by Maximum Likelihood with IQ-Tree 2 software (Minh et al. 2020), selecting the best-fit model of GTR+F+G4 with 2000 bootstraps. The tree file was visualised and annotated on iTOL (https://itol.embl.de/) (Ivica and Peer 2021). All the sequences accession numbers were listed in Table 1.

**Table 1.** Vouchers of specimens and GenBank accession number.

Taxon	Voucher	Accession no.
**Ingroup taxon**		
**sect.Diffusae (W.Becker) C.J.Wang**		
**ser.Australasiaticae Okamoto**		
*V.mucronulifera* Hand.-Mazz.	Lingyun, Guangxi, Zhou J S 311 (IBSC)	FJ002910
*V.sumatrana* Miq.	Lvchun, Yunnan, Wang Zheng-wei et al.WZW04206 (CSH)	OM406231
*V.kwantungensis* Melchior	Guidong, Hunan, Huang Cun-zhong LXC01887 (CSH)	OM406227
*V.kwantungensis* Melchior	Jinyunshan, Chongqing, Huang Yan-shuang HYS210206	OM406230
*V.kwangtungensis* Melchior	Malipo, Yunnan, Wang Zheng-wei et al. WZW04187 (CSH)	OM618008
*V.austrosinensis* Y.S.Chen & Q.E.Yang	Tianlin, Guangxi, Li Xiao-chen et al. LXC02318 (CSH)	OM406228
*V.davidii* Franch.	Mt. Maoershan, Guangxi, Zhou J S 273 (IBSC)	FJ002902
*V.davidii* Franch.	Mt. Jiulongshan, Zhejiang, Zhong Xin et al. ZX01824 (CSH)	OM406229
*V.grandisepala* W.Becker	Mt. Emeishan, Sichuan, Zhou J S 425 (IBSC)	FJ002903
*V.fargesii* H.Boissieu originally published as *V.principis*	Ruyuan, Guangdong, Zhou J S 103 (IBSC)	FJ002904
*Viola* sp. nov.	Maolan, Guizhou, Li Xiao-chen et al. LXC00927 (CSH)	OM406226
*Viola* sp. nov.	Maolan, Guizhou, Li Xiao-chen et al. LXC00323 (CSH)	OM406225
*Viola* sp. nov.	Maolan, Guizhou, Li Xiao-chen et al. LXC00324 (CSH)	OM406224
**ser.Diffusae (W.Becker) Steenis**		
*V.nanlingensis* J.S.Zhou & F.W.Xing	Mt. Nankunshan, Guangdong, Liang G. X. 0185 (IBSC)	FJ002916
*V.yunnanensis* W.Becker & H.Boiss.	Mt. Diaoluoshan, Hainan, Zhou J. S. *s.n.* (IBSC)	FJ002915
*V.diffusa* Ging	Huaiji, Guangdong, Gong Q. 00043 (IBSC)	FJ002917
*V.lucens* W.Becker	Tanziyan, Guizhou, Zhou J. S. 348 (IBSC)	FJ002913
*V.guangzhouensis* A.Q.Dong	Conghua, Guangdong, Dong A. Q. 1104 (IBSC)	FJ002918
**sect.Chamaemelanium Ging**		
*V.biflora* L.	–	FJ002905
*V.orientalis* (Maximowicz) W.Becker	–	FJ002909
*V.delavayi* Franch.	Diqing, Yunnan, Zhou J. S. Xing F. W. 487 (IBSC)	FJ002908
**sect.Viola L.**		
*V.collina* Bess.	–	FJ002880
*V.mirabilis* L.	–	MK828568
*V.rupestris* F.W.Schmidt	–	HM851448
*V.grypoceras* A.Gray	Mt. Lushan, Jianghxi, Liang G. X. 0002 (IBSC)	FJ002881
*V.acuminata* Ledeb.	–	FJ002884
**sect.Violidium (K. Koch) Juz.**		
*V.inconspicua* Blume	SCBG, Guangdong, Liang G. X. 0187 (IBSC)	FJ002897
*V.japonica* Langsdorff ex Candolle	–	EU591965
*V.prionantha* Bunge	Jinan,Shandong, Zhang R. J., Xing F. W. 17955 (IBSC)	FJ002893
*V.hancokii* W.Becker	–	FJ002890
*V.pekinensis* (Regel) W.Becker	–	FJ002892
*V.chaerophylloides* (Regel) W.Becker	–	FJ002898
*V.dissecta* Ledeb.	–	FJ002891
*V.magnifica* C. J. Wang & X.D.Wang	Mt. Lushan, Jiangxi, Liang G. X. 0038 (IBSC)	FJ002899
**sect.Bilobatae (W.Becker) Juz.**		
*V.verecunda* A.Gray	Mt. Nanling, Guangdong, Zhou J. S. 1553 (IBSC)	FJ002911
*V.triangulifolia* W.Becker	Mt. Jinggangshan, Jiangxi, Zhou J. S. 140 (IBSC)	FJ002912
**outgroup taxon**		
*Afrohybanthusenneaspermus* (L.) Flicker	–	HM483598

## ﻿Results

### ﻿Molecular Analysis

**Figure 1.**
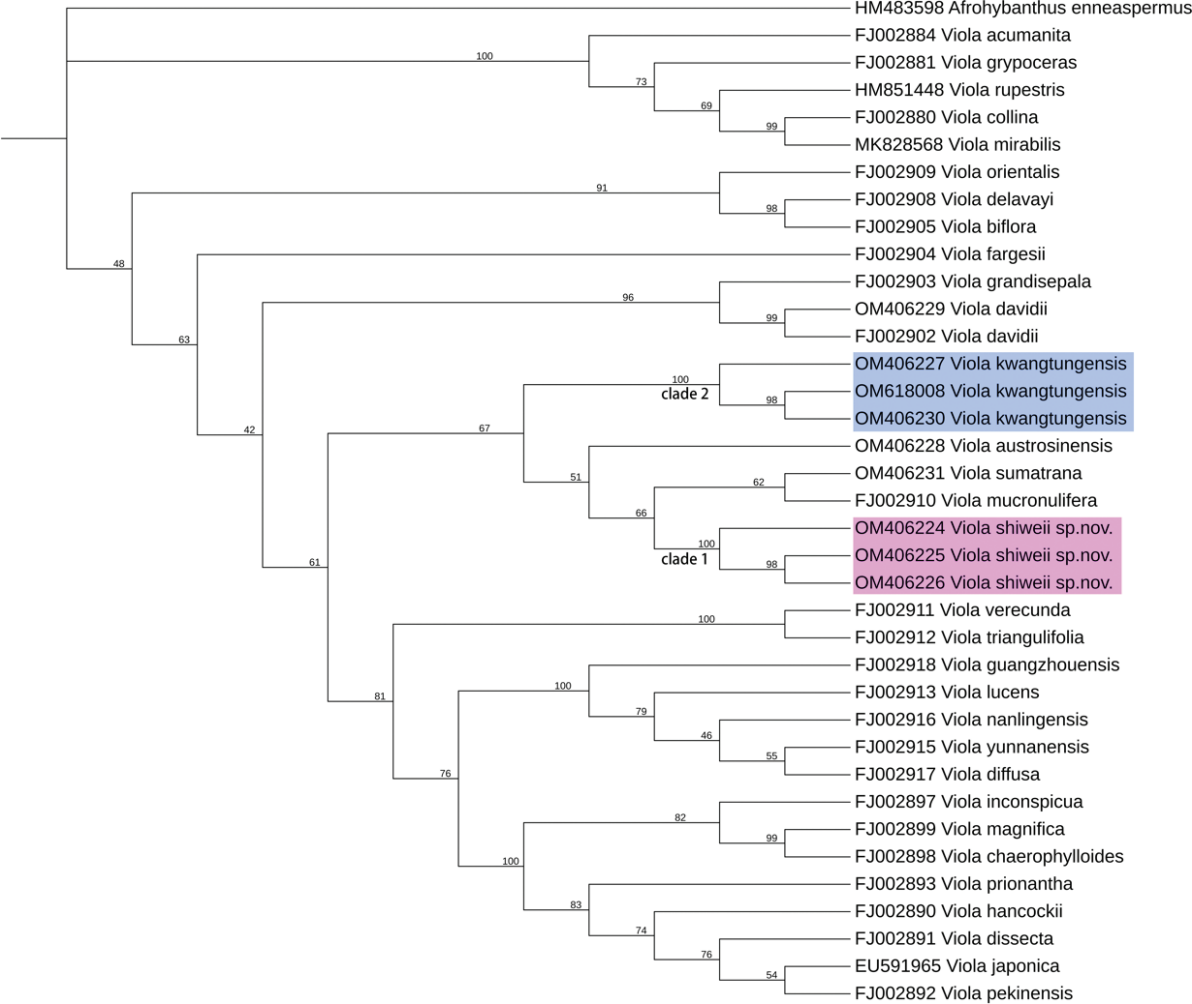
ML tree of the new species *Violashiweii* sp. nov. and its related species inferred by IQ-Tree 2, based on ITS dataset. Bootstrap values of the Maximum Likelihood are shown along the branches. Outgroup taxon: *Afrohybanthusenneaspermus*.

The ITS dataset comprises 37 accessions representing 32 species, including *Afrohybanthusenneaspermus* (L.) Flicker selected as an outgroup. The aligned matrix of ITS sequences was 696 bp in total. The result of ML is shown in Fig. 1. The samples of the putative new species (pink clade) clustered into a strongly supported monophyletic lineage (clade 1), forming a weak sister relationship with a clade formed by *V.mucronulifera* and *V.sumatrana*. The morphologically most similar *V.kwangtungensis* was resolved on a more distant phylogenetic position (clade 2, blue clade). Based on morphological characters and phylogenetic results, we recognise this unfamiliar violet as a distinct species and described it here as *Violashiweii* Xiao C. Li & Z.W.Wang.

### ﻿Taxonomic treatment

#### ﻿ Viola shiweii

Taxon classificationPlantaeLepidopteraHesperiidae

Xiao C. Li & Z. W. Wang sp. nov.

2EEA7109-CB24-54DD-9C0A-9C30C75088C3

urn:lsid:ipni.org:names:77297809-1

Figs 2 , 3 , Appendix 1 : Figs A1 , A2 , A3 , A4 , A5 , A6 , A7 , A8 , A9 , A10 , A11 , A12

##### Type.

**China.** Shanghai Chenshan Botanical Garden, cultivated plants collected from Guizhou, Qiannan Buyi and Miao Autonomous Prefecture (黔南布依族苗族自治区), Libo county (荔波县), Maolan National Nature Reserve (茂兰国家级自然保护区), on the rocks along the karst forest margin, 25°16'39.1039"N, 107°55'2.7598"E, 867 m elevation, 9 Nov 2019, Wang Zheng-wei and Li Xiao-chen, LXC00927 ***Holotype***: CSH0182173 (CSH!); ***isotypes***: ZJFC!, CSFI!, IBSC!.

**Figure 2.**
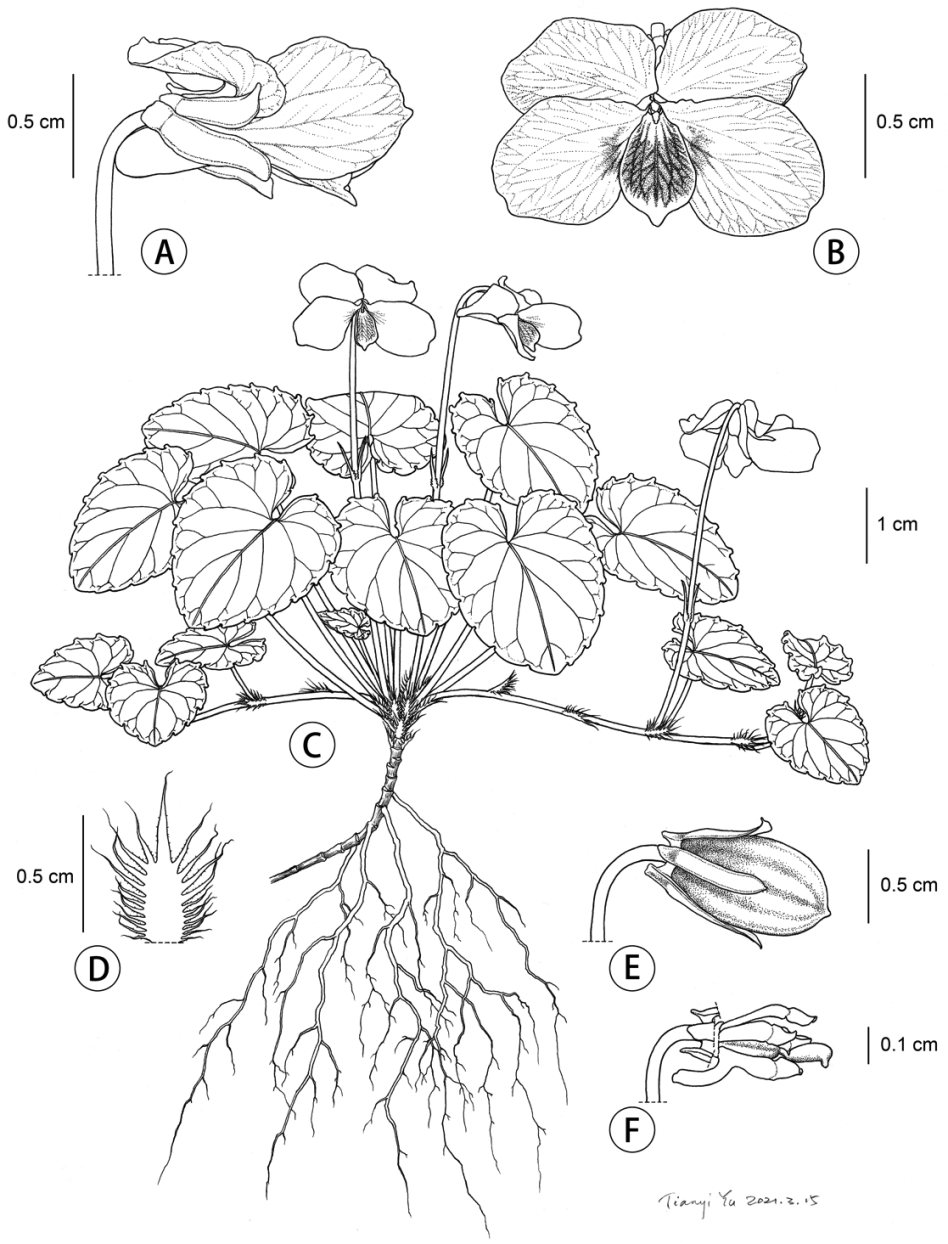
*Violashiweii* sp. nov. **A** flower side view **B** flower front view **C** habit **D** stipule **E** capsule **F** stamens and pistil.

##### Diagnosis.

The new species is morphologically most similar to *V.kwangtungensis* Melchior in its glabrous lateral petals and stoloniferous habit, but can be distinguished by its obtuse teeth along the leaf margin, its abaxially pale green leaf blade and its broader and entire sepals with a distinct basal appendage.

##### Description.

Perennial herb, acaulescent, with stolons. Rhizome short, straight or oblique, densely noded, usually covered by brown remains of stipules. **Stipules** free, brown, broadly lanceolate, 5–10 mm long, margin long fimbriate-dentate, lobes remotely dentate. **Basal leaves** glabrous, slightly grooved, with petioles stout, petioles unequal in length; blade thick leathery, ovate or suborbicular, 15–30 × 15–20 mm, base deep cordate, apex usually obtuse, abaxially green, scabrous, abaxially greyish-green, mid-vein distinct above, glabrous on both surfaces, margin glandular-serrate or shallowly glandular-crenate, slightly wavy, teeth obtuse at the apex; **stolon leaves** scattered, smaller. Pedicel equal to or longer than petiole, two bracts narrowly lanceolate, at the middle or lower part of the pedicel. **Sepals** 5, ca. 6 mm long and 2 mm wide, lanceolate, glabrous, margin narrowly membranous, apex somewhat acute, base distinctly decurrent, apex obtuse or shallowly dentate. Flower 1.5–2.5 cm in diameter, **petals** 5, white, posterior and lateral ones obovate, ca. 1.2 cm × 5 mm, narrow at the base, lateral petals purplish near the middle, glabrous, anterior petal shorter, ca. 10 mm (spur included) long, oblong, purple-veined, apex rounded, obtuse, spur saccate, 2–3 mm long and 1.5 mm wide. **Style** clavate, base geniculate, stigmas flattened on top, narrowly margined on lateral sides and abaxially, shortly beaked ventrally. **Capsule** ellipsoid, valves carinate, ca. 10 mm long and 2.5 mm wide, glabrous. **Seeds** black, ca. 2 mm long and 1 mm in diameter.

**Figure 3.**
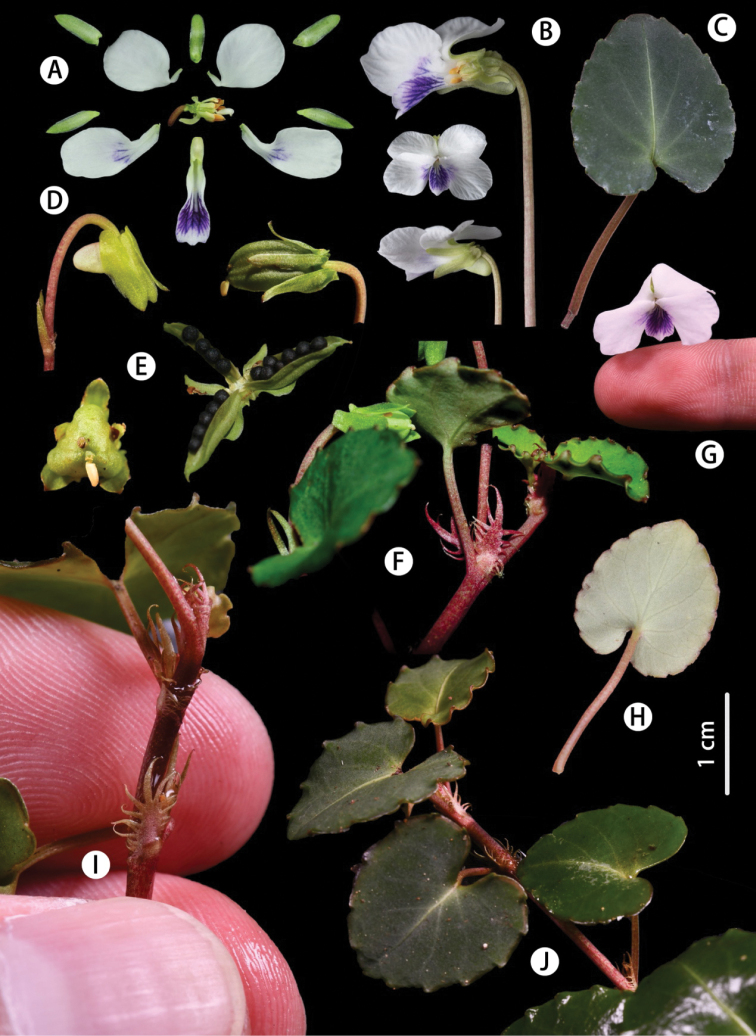
*Violashiweii* sp. nov. **A** petals, sepals, stamens and pistils **B, G** flower in front view and detail of a longitudinal section in side **C, H** basal leaf adaxially and abaxially **D** cleistogamous flowers **E** capsule and seeds **F** margin teeth **I** stipule **J** leaves on stolons.

##### Distribution and habitat.

The species was observed to grow on dry and partially shaded limestone, around the karst forest edge, at 700–900 m elevation.

##### Additional specimens examined.

**China**, Guizhou, Qiannan Autonomous Prefecture, Libo County, Maolan National Nature Reserve, karst forest, 24 Jul 2008, Zhang Dai-Gui 080724077 (JIU!); **China**, Guizhou, Qiannan Buyi and Miao Autonomous Prefecture, Libo County, Maolan National Nature Reserve, 21 Nov 2021, Li Xiao-chen, Wang Zheng-wei & Wei Hong-jin, LXC02320 (CSH!), LXC02322 (CSH!), LXC02323 (CSH!), LXC02324 (CSH!), LXC02325 (CSH!).

##### Phenology.

Cultivated plants flower in September-March, fruiting in September.

##### Etymology.

The specific name epithet “*shiweii*” was proposed in memory of Deng Shi-wei (191?-1936), who dedicated his life to the exploration of the flora of Guizhou. The Chinese name is given as “世纬堇菜”.

##### Conservation status.

**Figure 4.**
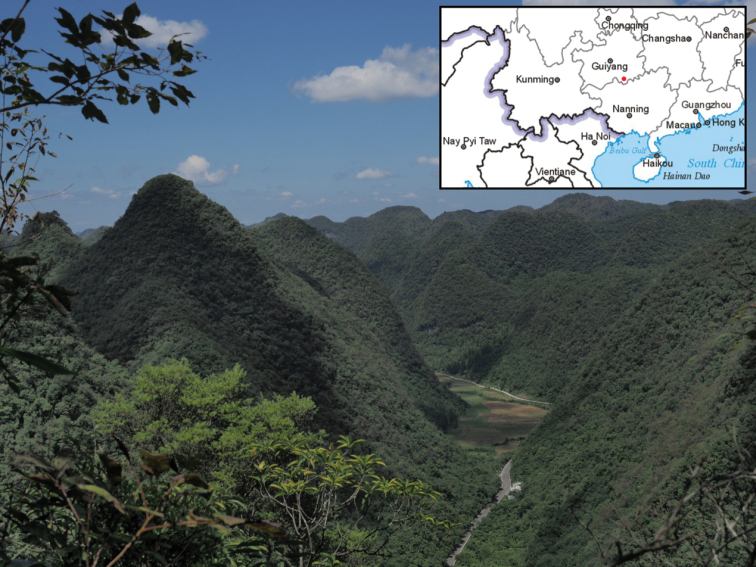
Habitat and distribution of *Violashiweii*.

Only two populations of *V.shiweii* are currently known from Maolan National Nature Reserve, Libo County, in an area of the karst formation across Guizhou and Guangxi (Fig. 4). This species is represented by no more than 200 large and mature individuals. Due to its rarity, the low number of individuals and habitat vulnerability, *V.shiweii* is considered to be Critically Endangered (CR, B1), according to the IUCN (2019).

## ﻿Discussion

Although our phylogenetic analysis, based on ITS sequences, did not fully clarify the infrageneric relationships within *Viola*, it produced informative evidence for differentiation amongst lower taxa. *V.shiweii* can be placed in Violaser.Australasiaticae (Okamoto et al. 1993), which is characterised by the stolons with scattered leaves, absent aerial stems, short spur of anterior petal and stigma beaked ventrally. The phylogenetic analysis in this study (Fig. 1) also confirmed this conjecture (Fig. 1); however, the monophyly of Violaser.Australasiaticae was not supported, which was consistent with a previous study (Gong et al. 2010). Violaser.Australasiaticae was proved to be nested in the subg. Violasect.Plagiostigma Godr. (Marcussen et al. 2012).

Violaser.Australasiaticae comprises ca. 27 species, widely distributed in the Himalayan Region, southern China, south-eastern Asia and Ryukyu Island of Japan, with 14 species occurring in China (Chen 2006), in which *Violadavidii*, *V.schneideri* W.Becker, *V.kwangtungensis*, *V.mucronulifera* and *V.austrosinensis* form a complex in this series and caused mass misidentification due to their high degree of morphological similarity.

*Violadavidii* Franchet was published by Adrien René Franchet (1885), based on the collection of David from Moupine (Baoxing County, Sichuan, China) (isotype: David#s.n., K000254222) in 1869 [1870]. It is a morphologically variable and widespread species characterised by its ovate or ovate-orbicular leaf blade with 6–8 rounded teeth along each side, bearded lateral petals and short spurred anterior petal. It was originally regarded as species similar to *V.biflora* L., but its beaked stigma (vs. bilobed), white and purple petals (vs. yellow) indicated a distinctly different affiliation amongst the genus. Later, Becker (1921) described a strikingly similar violet with ovate leaves, *V.schneideri* W.Becker, based on the collection of Schneider (isotype: Schneider C.K. #739, G00343327) from Te-chang (De-chang County, Sichuan, China), but the diagnostic leaf shape falls within the morphological variation of *V.davidii* Franchet and, for this reason, it was recently treated as its synonym, which is further supported by overlapping distributions (Chen 2006). The only collection of *V.shiweii* before this study was misidentified as *V.davidii*. *Violashiweii* shares a similar leaf shape with *V.davidii*, but can be differed by its glabrous lateral petals and obtuse teeth along the leaf margin.

**Figure 5.**
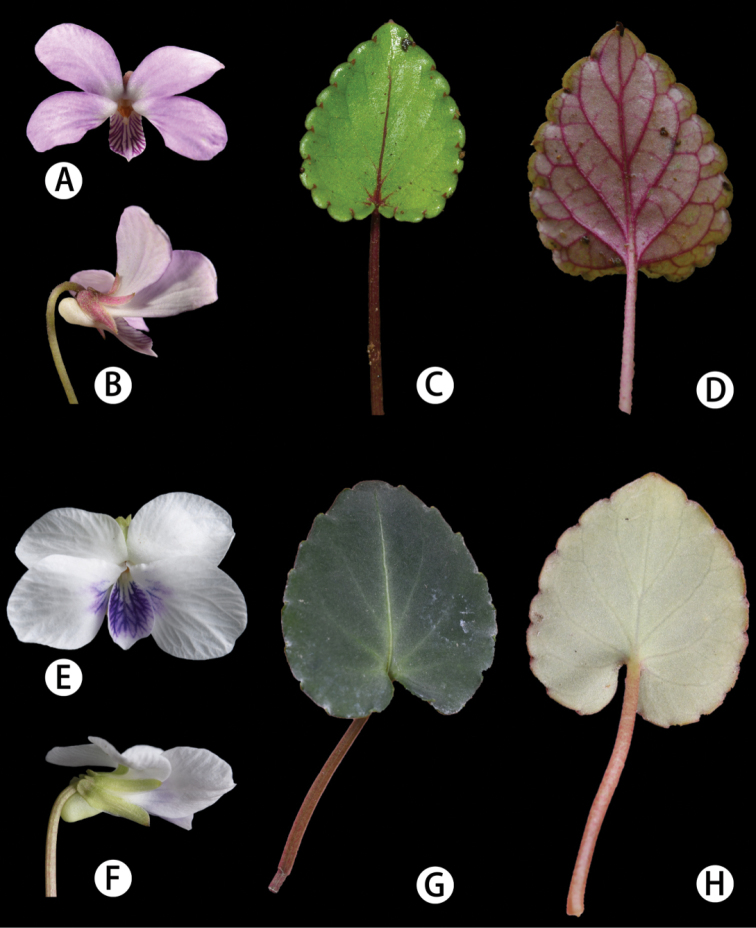
Flowers and leaves of *Violashiweii* sp. nov. and *Violakwangtungensis***A, B***V.kwangtungensis*, lower, front and side views **C, D***V.kwangtungensis*, adaxial and abaxial leaf surfaces **E, F***Violashiweii*, flower, front and side views **G, H***V.kwangtungensis*, adaxial and abaxial leaf surfaces.

**Figure 6.**
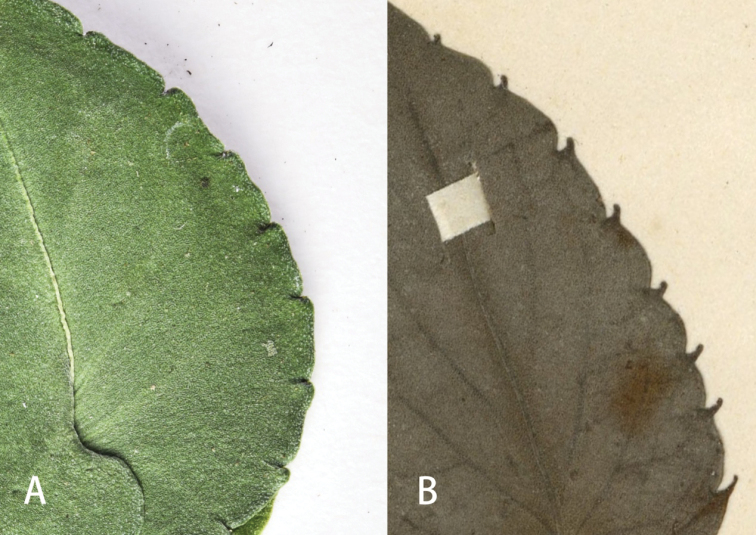
Leaf margin of *V.shiweii* sp. nov. and *V.kwangtungensis***A***V.shiweii*, holotype **B***V.kwangtungensis*, isotype: P. Ko #50326, A00067196.

*Violakwangtungensis* Melchior, which shows the highest resemblance to *V.shiweii* (Table 2, Figs 5, 6) is an overlooked species frequently being confounded with *V.davidii* or *V.mucronulifera*, but considered as a distinct taxon by Flora of China (Chen et al. 2007). *Violamucronulifera* Hand.-Mazz. was published by Handel-Mazzetti (1931), based on the collection of R. C. Ching #7016 from Guangxi (holotype: PE00025463, isotypes: NY00097644 & A00067198) and is characterised by the distinctly stipitate tooth glands. Later, *V.kwangtungensis*Melchior (1933) was published, based on a collection of Woon-Young Chun and his assistants (isotype: P. Ko #50326, A00067196) from Guangdong, which can be easily recognised by its characteristic leaf crenation of the leaves, but it was subsequently reduced to a synonym of *V.mucronulifera* in Flora Reipublicae Popularis Sinicae (Wang 1991). *Violakwangtungensis* has spinules at the apex of the teeth, as the horizontal extension of the teeth, while the spinules of *V.mucronulifera* are perpendicular to the leaf blade and placed between the teeth, which can be distinguished in field observation. *Violakwangtungensis* also used to be considered conspecific with *V.schneideri* due to morphology transition in the spinose, based only on the specimen observation (Zhou et al. 2008b).

**Table 2.** Morphology and distribution comparison between *Violashiweii* sp. nov., *V.kwangtungensis* and *Violadavidii*.

	*Violashiweii*	*Violakwangtungensis*	*Violadavidii*
Leaf blade	Ovate or orbicular, apex usually obtuse, base deep cordate, greyish-green abaxially.	Ovate to triangularly ovate, base shallowly cordate, apex usually acute, purple abaxially.	Ovate or ovate-orbicular, glaucous abaxially, base deeply coedate, apex rounded or acute.
Leaf margin	Serrate	Crenate	Shallowly 6–8-crenate on each side.
Stipule	Long fimbriate	Fimbriate	Remotely fimbriate-dentate
Sepals	Lanceolate, ca. 6 mm × 2 mm, entire, green, glabrous, basal distinctly decurrent.	Lanceolate, 3–5 mm × 1.5–2 mm, sparsely shallowly dentate, purplish-red, sparsely pubescent, base not decurrent.	Lanceolate or ovate-lanceolate, 5–6 mm × 1.5–2 mm, brown, glabrous, base shortly decurrent, margin narrowly membranous, apex truncate
Posterior and lateral petals	Obovate, base constricted	Obovate-oblong	Oblong-obovate
Seed	Black	Brown	Brown
Habitat	Dry and partially shaded limestone	Humid and shaded stream valley	Shaded place under forest, stream valley, or grassy slope.
Distribution	Guizhou, Guangxi.	Fujian, N Guangdong, Guizhou, Hunan, Jiangxi, Sichuan, Yunnan, and Taiwan (Lu et al. 2019)	Chongqing, Fujian, Guangdong, Guangxi, Guizhou, Hubei, Hunan, Jiangxi, Sichuan, SE Xizang, Yunnan, Zhejiang.

More recently, as the latest supplement of this complex, a new species, *V.austrosinensis*, distinguished from *V.kosanensis* Hayata (ser. Rosulantes Borbas (Y.S.Chen)), was described, of which the leaves were coriaceous, glabrous, not glandular-dotted on the abaxial surface (Chen and Yang 2008). *Violaaustrosinensis* is different from *V.shiweii* in its ovate leaf blade and acute anterior petal’s apex.

In China, *V.mucronulifera* was found to occur only in the Province of Yunnan and its type locality in Guangxi; its occurrence in Guizhou was a mistake caused by the misidentification of *V.kwangtungensis* in Flora of Guizhou (Yao 1989), as we personally observed in the locality cited in this work (Fanjingshan, Jiangkou). The morphology and distribution differences between *V.shiweii*, *V.kwangtungensis* and *V.davidii* are listed in Table 2. Comparision of V.shiweii and V. kwangtungensi was visualised in Figs 5, 6. Keys to *V.shiweii* and its allies were also presented.

### ﻿Keys to *Violashiweii* sp. nov. and its related species (ser. Australisiaticae) in China

**Table:** 

1	Spur 5–7 mm, anterior petal 2-lobed at apex	***V.formosana***
–	Spur shorter than 5 mm, anterior petal rounded, obtuse or acute at apex	**2**
2	Stipules usually entire, sepals broad ovate, ca. 5 mm wide	***V.grandisepala***
–	Stipules fimbriate, sepals lanceolate, much narrower, not more than 5 mm	**3**
3	Leaf blade spinulose along margin	**4**
–	Leaf blade without spinules along margin	**5**
4	Leaves conspicuously spinose between teeth	***V.mucronulifera***
–	Leaves shortly spinose at apex of teeth	***V.kwangtungensis***
5	Leaves ovate, orbicular or nearly orbicular, apex obtuse	**6**
–	Leaves cordate or oblong-ovate, apex acuminate	**10**
6	Lateral petals beard at base	***V.davidii***
–	Lateral petals glabrous at base	**7**
7	Leaves serrata, teeth have obtuse apices, apex of anterior petal obtuse	***V.shiweii***
–	Leaves crenate, without obtuse teeth along margin	**8**
8	Leaves coriaceous, base shallowly cordate, anterior petal acute	***V.austrosinensis***
–	Leaves chartaceous, orbicular or nearly orbicular, base deeply cordate, anterior rounded	**9**
9	Leaves adaxially scabrous, sparsely pubescent	***V.duclouxii***
–	Leaves adaxially shiny, glabrous	***V.sikkimensis***
10	Rhizome short, densely noded	**11**
–	Rhizome nodes elongated and stout	**12**
11	Leaves glabrous, shiny adaxially	***V.nitida***
–	Leaves densely pubescent	***V.fargesii***
12	Plant densely pubescent	***V.yunnanensis***
–	Plant glabrous or sparsely pubescent	**13**
13	Leaves blade glabrous, sepals ovate	***V.nuda***
–	Leaves more or less pubescent, sepals lanceolate	**14**
14	Leaves and capsules dot-like brown glandular, lateral petals glabrous	***V.sumatrana***
–	Leaves not glandular, lateral petals bearded	***V.thomsonii***

## Supplementary Material

XML Treatment for Viola shiweii

## Figures and Tables

**Figure A1.**
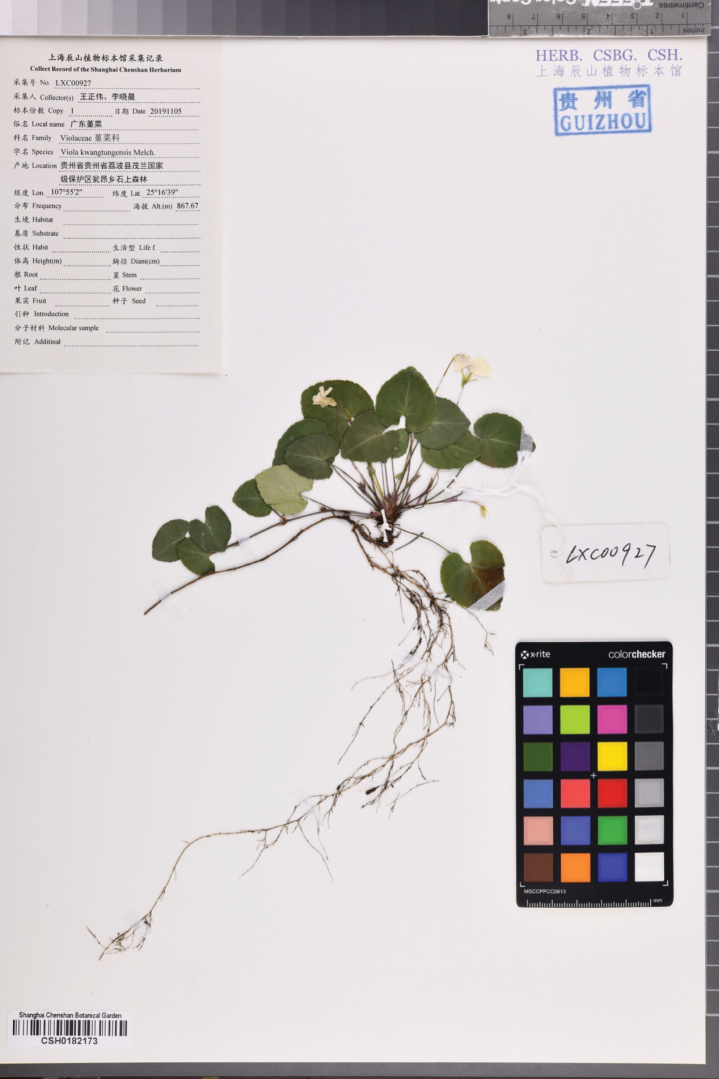
Holotype: LXC00927 (CSH0182173).

**Figure A2.**
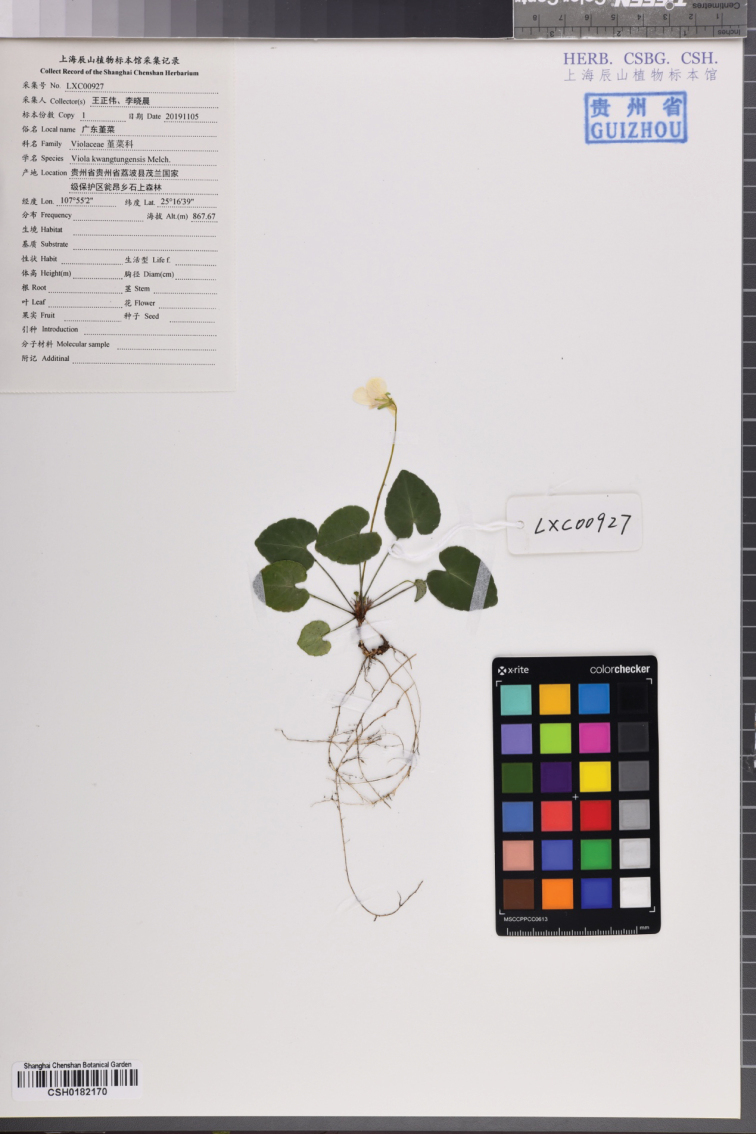
Isotype: LXC00927 (CSH0182170).

**Figure A3.**
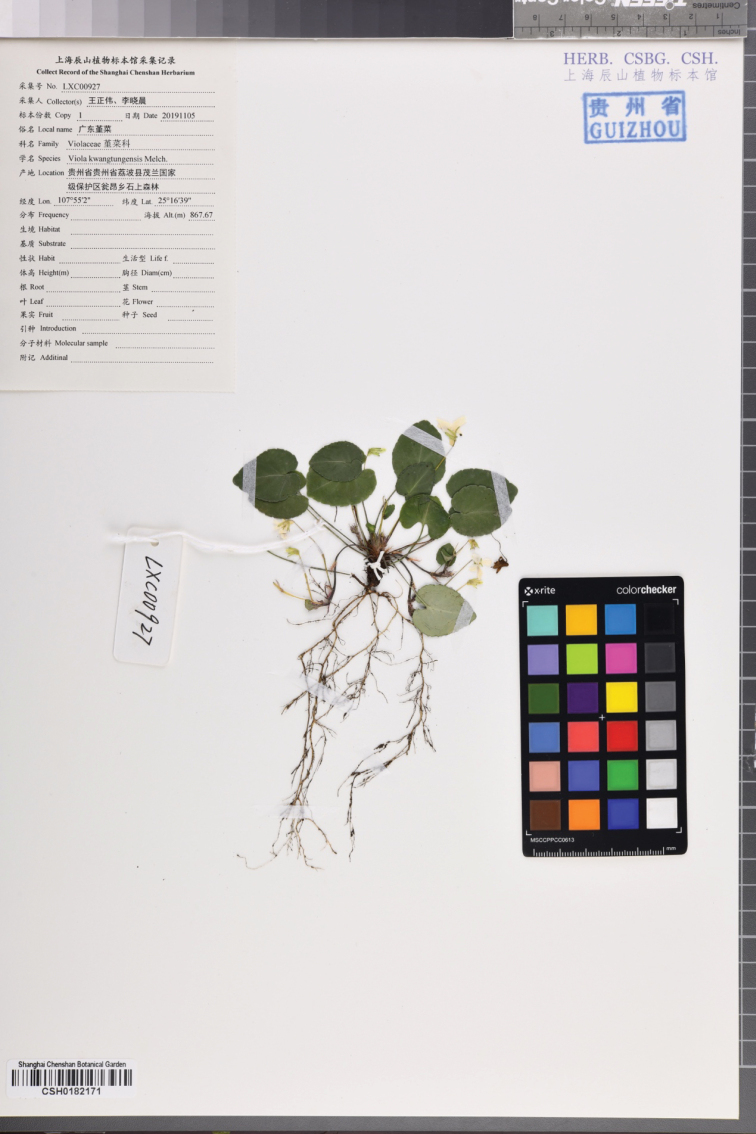
Isotype: LXC00927 (CSH0182171).

**Figure A4.**
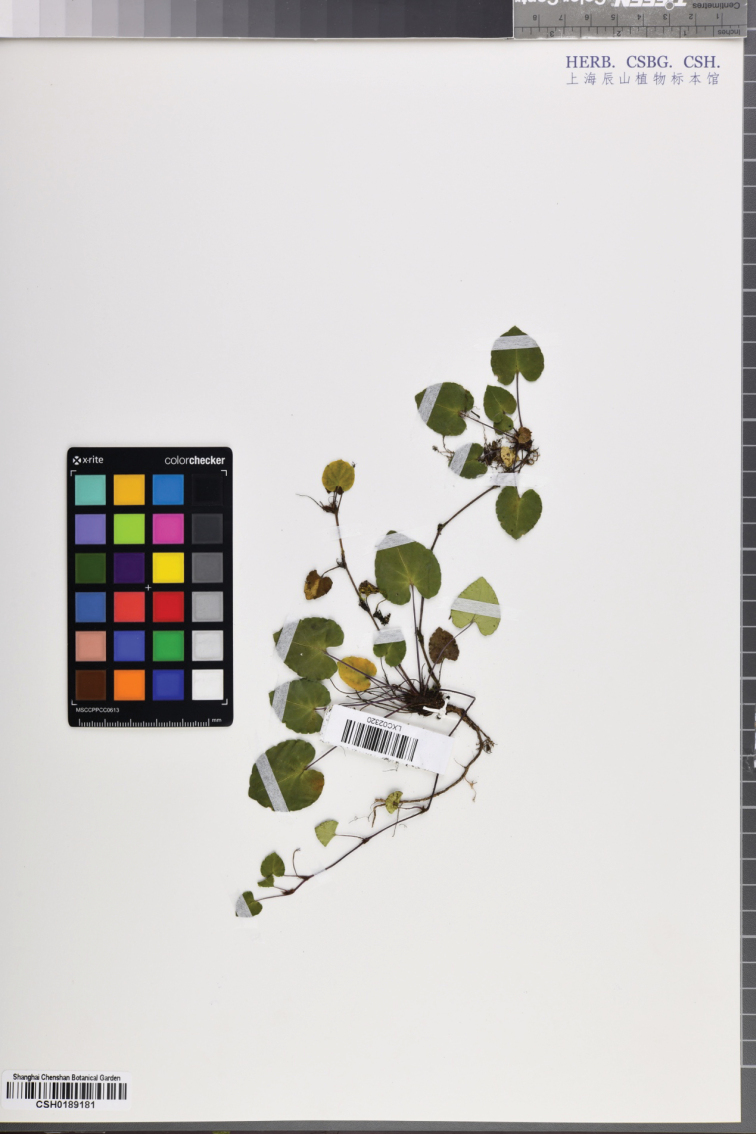
Isotype: LXC00927 (CSH0182172).

**Figure A5.**
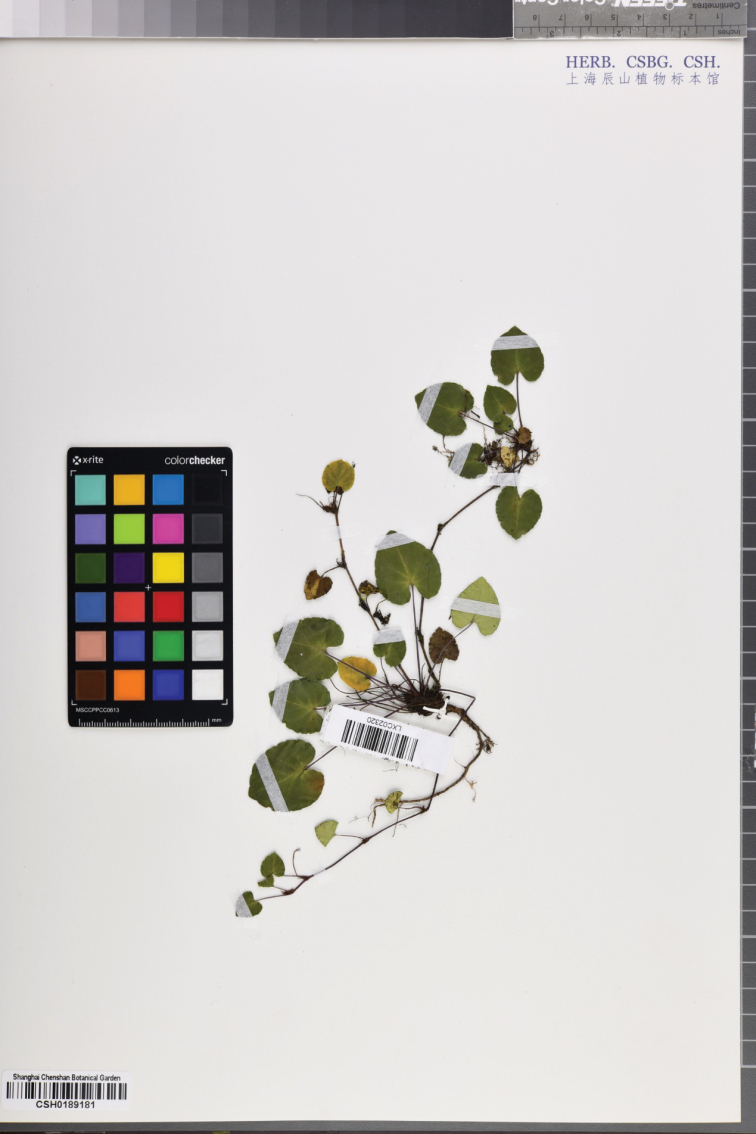
Paratype: LXC02320 (CSH0189181).

**Figure A6.**
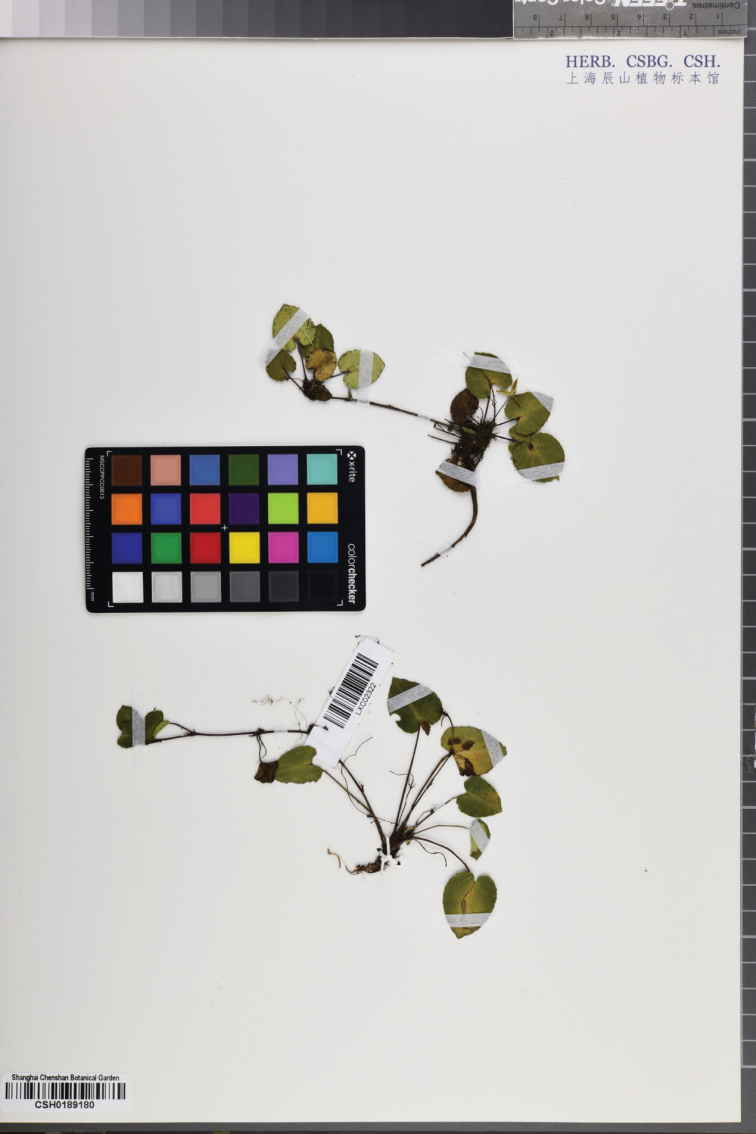
Paratype: LXC02322 (CSH0189180).

**Figure A7.**
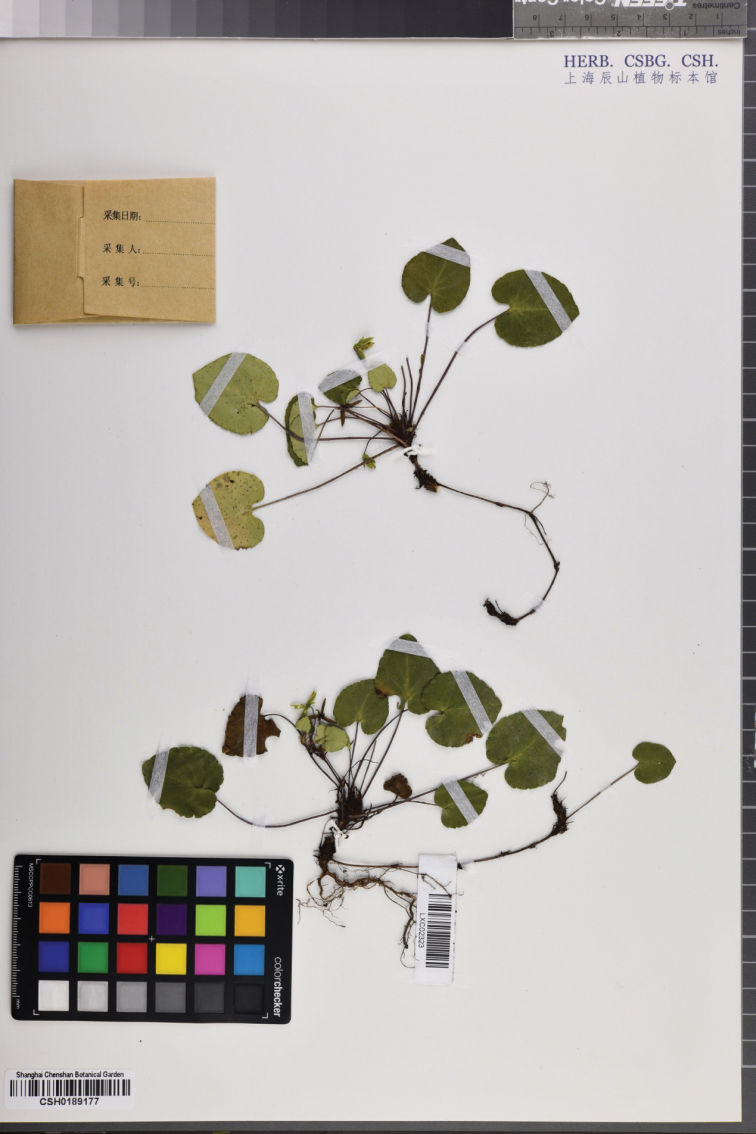
Paratype: LXC02323 (CSH0189177).

**Figure A8.**
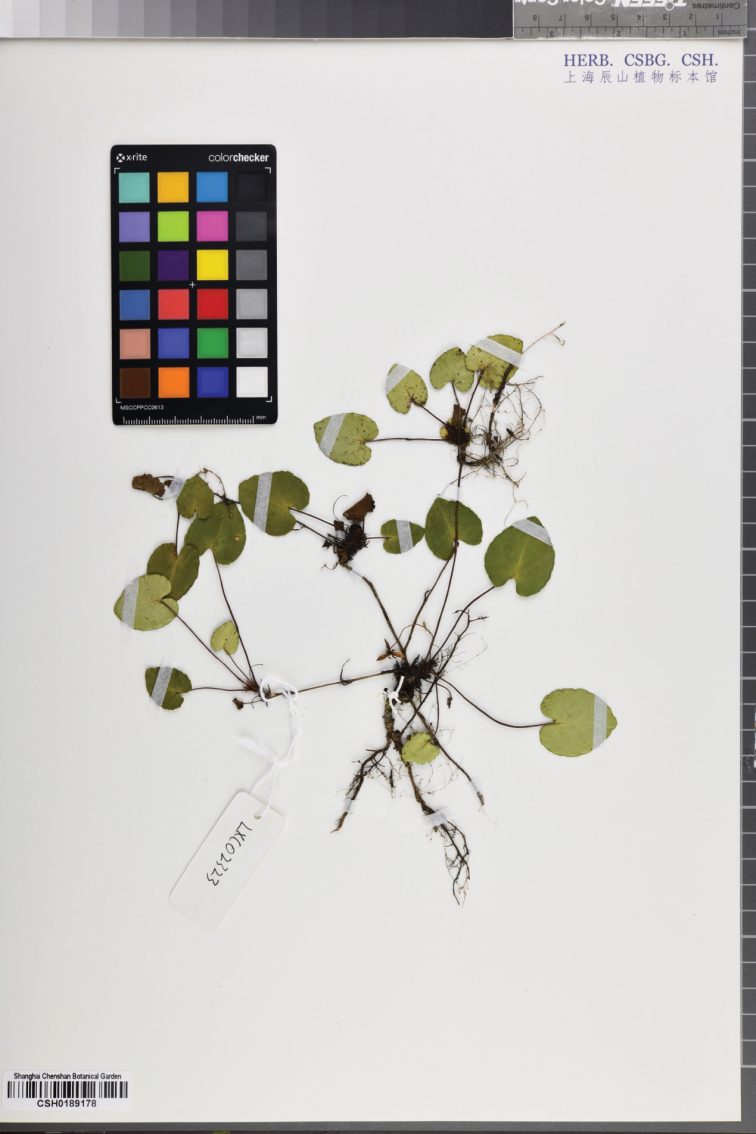
Paratype: LXC02323 (CSH0189178).

**Figure A9.**
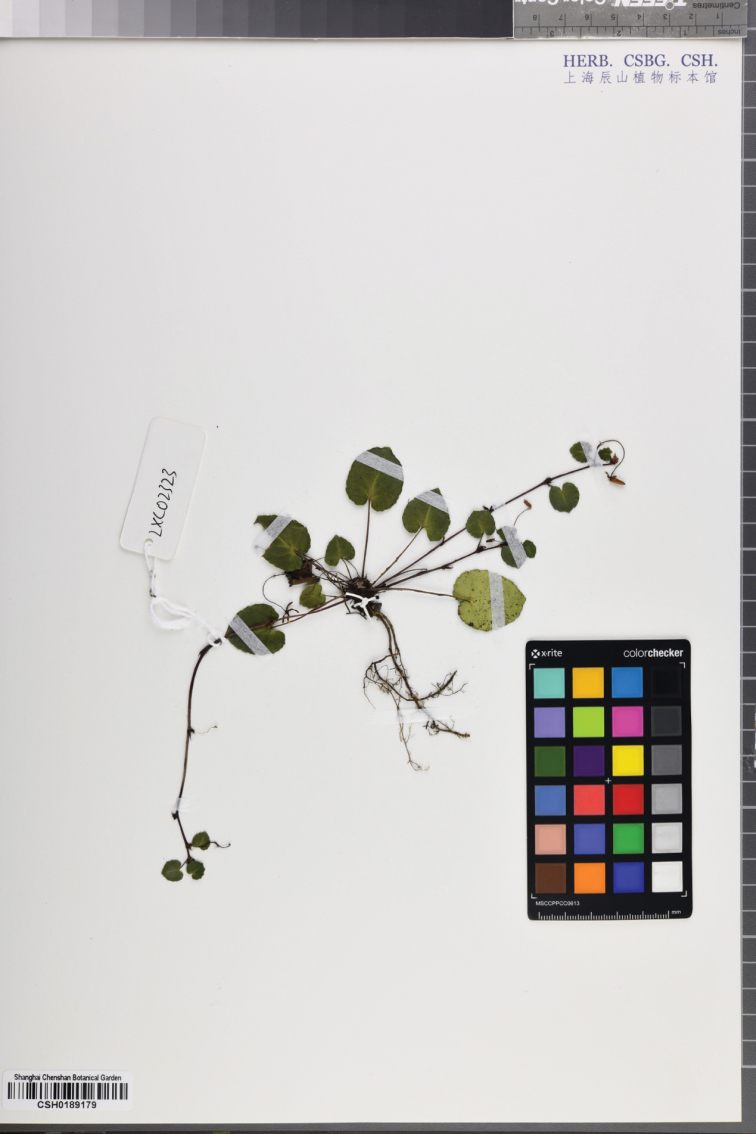
Paratype: LXC02323 (CSH0189179).

**Figure A10.**
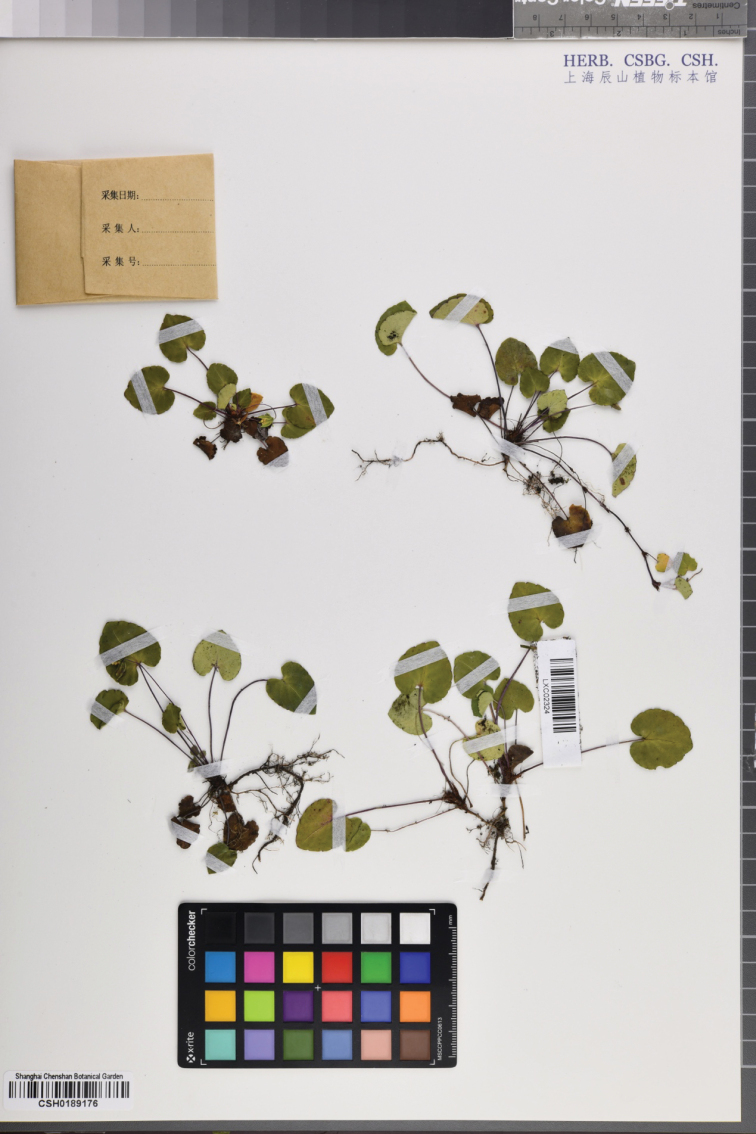
Paratype: LXC02324 (CSH0189176).

**Figure A11.**
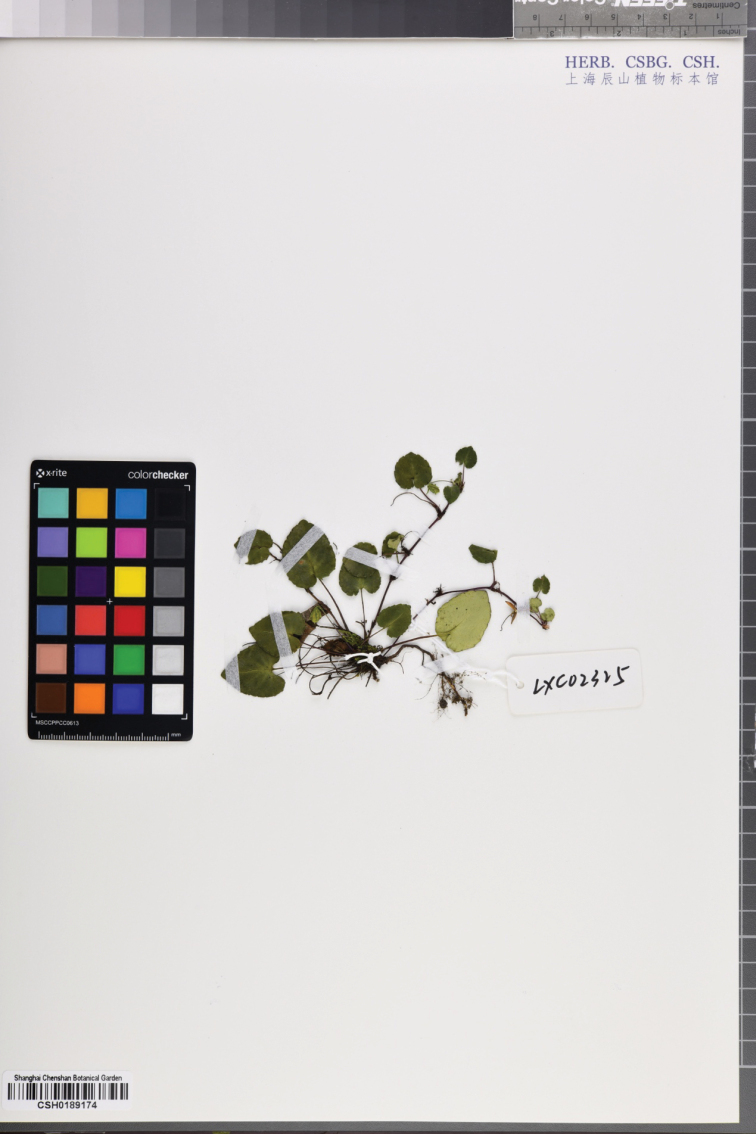
Paratype: LXC02325 (CSH0189174).

**Figure A12.**
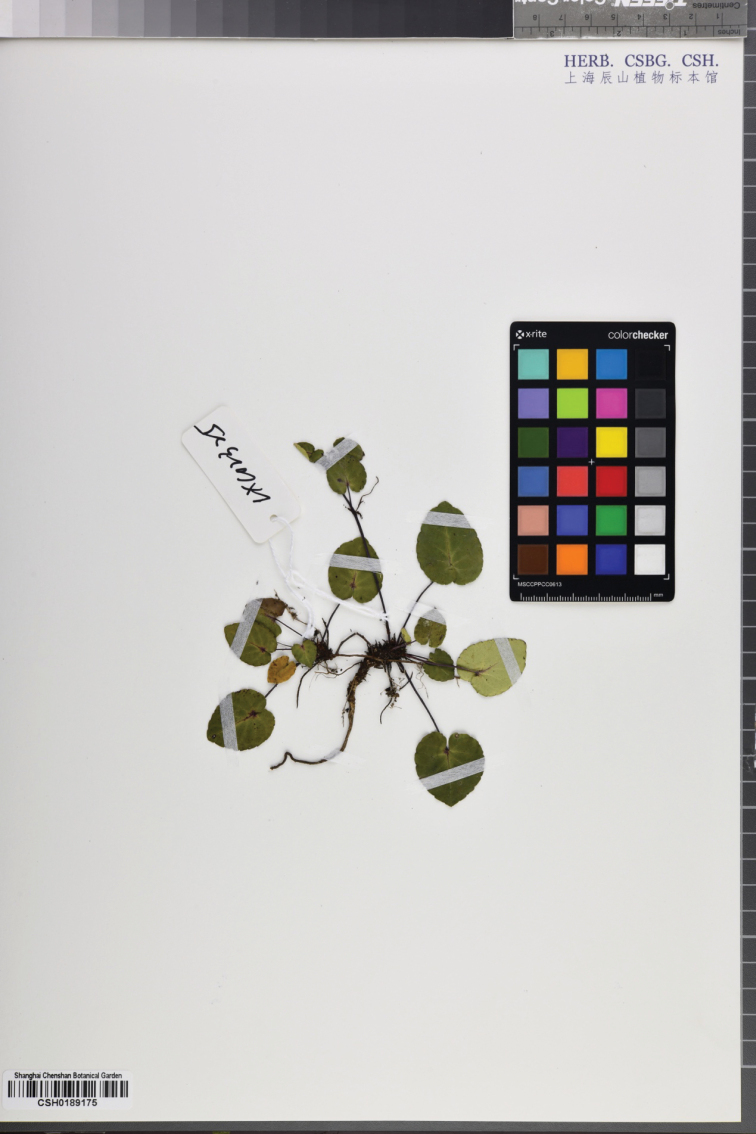
Paratype: LXC02325 (CSH0189175).
